# The Critical Assessment of Oxidative Stress Parameters as Potential Biomarkers of Carbon Monoxide Poisoning

**DOI:** 10.3390/ijms241310784

**Published:** 2023-06-28

**Authors:** Piotr Hydzik, Renata Francik, Sławomir Francik, Ewa Gomółka, Ebru Derici Eker, Mirosław Krośniak, Maciej Noga, Kamil Jurowski

**Affiliations:** 1 Toxicology Clinical Department, University Hospital, Jagiellonian University Medical College, 31-008 Kraków, Poland; 2 Institute of Health, State Higher Vocational School, 33-320 Nowy Sącz, Poland; 3 Department of Bioorganic Chemistry, Faculty of Pharmacy, Jagiellonian University Medical College, 31-008 Krakow, Poland; 4 Department of Mechanical Engineering and Agrophysics, Faculty of Production and Power Engineering, University of Agriculture in Krakow, 31-103 Krakow, Poland; 5 Toxicological Information and Laboratory Analysis Laboratory University Hospital, Jagiellonian University Medical College, Jakubowskiego 2, 30-688 Kraków, Poland; 6 Faculty of Pharmacy, Mersin University, 33343 Mersin, Turkey; 7 Department of Food Chemistry and Nutrition, Faculty of Pharmacy, Jagiellonian University Medical College, 31-008 Krakow, Poland; 8 Department of Regulatory and Forensic Toxicology, Institute of Medical Expertises, 91-205 Łódź, Poland; 9 The Laboratory of Innovative Research and Analyzes, Institute of Medical Studies, Medical College, Rzeszów University, 35-310 Rzeszow, Poland

**Keywords:** poisoning, carbon monoxide, oxidative stress, treatment options

## Abstract

In conventional clinical toxicology practice, the blood level of carboxyhemoglobin is a biomarker of carbon monoxide (CO) poisoning but does not correspond to the complete clinical picture and the severity of the poisoning. Taking into account articles suggesting the relationship between oxidative stress parameters and CO poisoning, it seems reasonable to consider this topic more broadly, including experimental biochemical data (oxidative stress parameters) and patients poisoned with CO. This article aimed to critically assess oxidative-stress-related parameters as potential biomarkers to evaluate the severity of CO poisoning and their possible role in the decision to treat. The critically set parameters were antioxidative, including catalase, 2,2-diphenyl-1-picryl-hydrazyl, glutathione, thiol and carbonyl groups. Our preliminary studies involved patients (*n* = 82) admitted to the Toxicology Clinical Department of the University Hospital of Jagiellonian University Medical College (Kraków, Poland) during 2015–2020. The poisoning was diagnosed based on medical history, clinical symptoms, and carboxyhemoglobin blood level. Blood samples for carboxyhemoglobin and antioxidative parameters were collected immediately after admission to the emergency department. To evaluate the severity of the poisoning, the Pach scale was applied. The final analysis included a significant decrease in catalase activity and a reduction in glutathione level in all poisoned patients based on the severity of the Pach scale: I°–III° compared to the control group. It follows from the experimental data that the poisoned patients had a significant increase in level due to thiol groups and the 2,2-diphenyl-1-picryl-hydrazyl radical, with no significant differences according to the severity of poisoning. The catalase-to-glutathione and thiol-to-glutathione ratios showed the most important differences between the poisoned patients and the control group, with a significant increase in the poisoned group. The ratios did not differentiate the severity of the poisoning. The carbonyl level was highest in the control group compared to the poisoned group but was not statistically significant. Our critical assessment shows that using oxidative-stress-related parameters to evaluate the severity of CO poisoning, the outcome, and treatment options is challenging.

## 1. Introduction

In Poland, approximately 5800 hospital admissions are reported yearly due to carbon monoxide poisoning [1]. Carbon monoxide (CO) is a colorless and odorless poisonous gas produced by the incomplete oxidation of fossil fuels and carbonaceous organic compounds (e.g., coal, natural gas, wood, and kerosene) [2]. The most frequently reported cases of CO intoxication are unintentional poisonings, i.e., accidental. Most deaths occur due to malfunctioning or obstructed exhaust systems, weather changes, and poor ventilation, restricting outdoor airflow into a building (especially bathing in a non-ventilated bathroom and staying in the car with the engine running in a closed garage).

It is well known that CO reversibly binds to hemoglobin and shifts the oxyhemoglobin dissociation curve to the left. In this way, it decreases the oxygen-carrying capacity of the blood and interferes with the release of oxygen at the peripheral tissue level [3]. These two main mechanisms of action underlie the potentially toxic effects of low-level CO exposure. However, the principal cause of CO-induced toxicity at low exposure levels is believed to be increased carboxyhemoglobin (COHb) formation. Clinical observation shows blood COHb levels do not correlate well with clinical symptoms and patient status [4].

Furthermore, it is known that the sole effect of CO is not only to block oxygen transfer in the blood but also to bind to other extravascular proteins such as myoglobin and non-Hb hemoproteins such as cytochrome c oxidase and cytochrome P-450. Adaptable CO (which is involved in cellular adaptation to oxidative stress and vascular dysfunction, leading to the maintenance of cellular and vascular homeostasis) regulates mitochondrial function to generate reactive oxygen species (ROS), which are responsible for controlling cellular redox states and adaptive responses to oxidative stress [5]. Numerous experiments demonstrate that CO is involved in the adaptation of cells to oxidative stress leading to the maintenance of cellular homeostasis [6]. At physiological state, approximately 1 to 3% of the oxygen consumed is incompletely reduced to anion superoxide, quickly transformed into hydrogen peroxide by superoxide dismutase (SOD) located in the mitochondrial matrix. A possible model for CO action is the generation of mitochondrial ROS based on partially and/or reversely inhibiting cytochrome c oxidase (complex IV), leading to electron accumulation at the complex III levels, which facilitates anion superoxide generation [6]. On the other hand, at low levels of CO, it is possible to improve mitochondrial respiration [7]; It can be speculated that CO induces mitochondrial ROS generation due to accelerated oxidative phosphorylation.

Cytochrome prefers oxygen to CO by a factor of 9:1, which may explain the disparity between the blood level of COHb and the clinical effects of poisoning and some beneficial effects of hyperbaric oxygen therapy (HBO). The biological half-life of carboxycytochrome c oxidase is not known. However, it may be an essential factor in the genesis of late CO poisoning sequelae and provides a rational basis for determining the duration of oxygen therapy. Other mechanisms of CO-induced toxicity have been hypothesized and evaluated, such as hydroxyl radical production and lipid peroxidation, in animals. However, as of yet, none have been demonstrated to operate at relatively low CO exposure levels [7].

The inhibition of energy coupling and aerobic metabolism violates the steady-state equilibrium between pro-oxidants and antioxidants. An imbalance in favor of pro-oxidants potentially leads to damage called oxidative stress [8]. CO-mediated ROS production initiates intracellular signal events, which regulate the expression of adaptive genes implicated in oxidative stress and function as a signaling molecule to promote vascular functions, including angiogenesis and mitochondrial biogenesis [6]. Animal studies indicate that low blood COHb levels can cause perivascular oxidative changes by releasing free radical nitric oxide from the vascular endothelium and platelet [9]. This also suggests the role of oxidative stress and hypoperfusion in CO toxicity. Organs most sensitive to CO toxicity are those with high blood flow and oxygen requirements, such as the brain, heart, and skeletal muscles. The most observed symptoms of acute CO poisoning are weakness, fatigue, malaise, headache, drowsiness, confusion, syncope, seizure, nausea, vomiting, vertigo, palpitation, and chest pain.

Prehospital care is generally limited to an interruption of CO exposure with 100% oxygen therapy with a non-rebreather mask. If the patient is in a deep comatose state and unstable, artificial ventilation intubation is necessary. Oxygen therapy reduces the biological half-time of COHb and increases oxygen concentration in the blood. This improves oxygen delivery to tissues and cells and reduces hypoxia. Most of the time, oxygen therapy is effective with the complete resolution of symptoms. In 10–30% of CO intoxication cases, delayed neuropsychiatric syndrome (DNS) could present with the most common manifestations such as cognitive and behavioral impairment, memory loss, movement disorders with parkinsonian features, seizures, visual impairment, depression, hallucinations, and urine incontinence [10,11,12,13,14]. The exact pathogenesis of DNS remains unknown. Increasing evidence indicates that the brain damage caused by CO intoxication results from mitochondrial oxidative stress in the central nervous system and the oxidation of proteins (the oxidation of thiol groups and the formation of carbonyl derivatives). These reactions lead to protein damage leading to abnormal immune response with white matter demyelination and leukoencephalopathy and the appearance of the delayed effects seen in DNS.

Generally, the severity of the poisoning is assessed after the end of the acute phase and the evaluation of organ damage caused by poisoning. In Poland, the Pach scale is applied for such an assessment [15,16]—Table 1. The Pach scale of CO poisoning (developed initially by Janusz Pach) consists of factors that influence and determine the severity of the poisoning. Each factor is classified from 0 to 3 points, and the total points indicate the severity of CO poisoning ranging from I° to III°.

Basic laboratory blood tests include evaluating biomarkers such as COHb and lactate blood levels, troponin level, creatine kinase (CPK), and aminotransaminase activity of aminotransaminases (e.g., AST and ASPAT) in the blood.

The multidirectional mechanism of CO toxicity releases ROS and reactive nitrogen species (RNS), significantly disrupting redox homeostasis. Several assays are available to measure oxidative stress. The basic parameters used to estimate the antioxidant status are glutathione (GSH), carbonyl groups (=CO), sulfhydryl (thiol) groups (–SH), 1,1-diphenyl-2-picryl hydrazyl radical (DPPH), catalase (CAT) and glutathione peroxidase (GPX activity). These parameters are not used routinely in clinical practice but could be suitable potential biomarkers to assess the severity of CO poisoning and predict poisoning complications. To date, no strategy is available to evaluate antioxidative parameters in the blood that could help select therapy and minimize oxidative stress in CO poisoning patients.

This work aimed to evaluate parameters related to oxidative stress, such as the activity and levels of selected antioxidative parameters (CAT, DPPH, GSH, –SH, =CO, CAT/GSH ratio and SG/GSH ratio) as potentially valuable biomarkers to assess the severity of CO poisoning from monoxide and their possible role in deciding treatment options.

## 2. Results and Discussion

Serum antioxidant parameters were evaluated for patients (*n* = 82) divided into four groups, i.e., the C control group, S-Pach scale = I°, II°, III°. The hypothesis of the influence of exposure time (factor) on the severity of poisoning (value of the Pach scale) was also evaluated. A one-way ANOVA test was performed for three groups (levels of factor: the Pach scale = I°, II°, III°), where the time of exposure to CO (T-CO) was adopted as a dependent variable based on an interview with an intoxicated patient.

The ANOVA results (Table 2) show a statistically significant effect of the independent variable—the Pach scale (S-Pach)—on seven dependent variables: CAT, DPPH, SH, GSH, CAT/GSH, SH/GSH, and T-CO. No statistically significant effect of S-Pach on =CO derivates was found. The results of antioxidant parameters for different groups are presented in Table 3. Data are presented as means from independent measurements ± standard deviation (SD).

**Figure 1:**
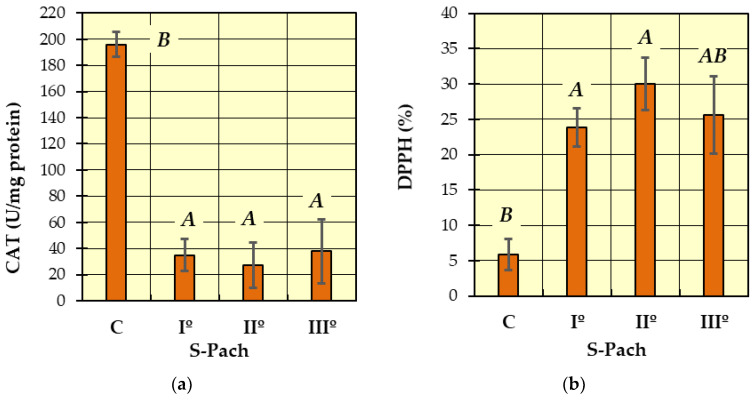
The values of measured antioxidative parameters in the serum of patients with CO poisoning (S-Pach = I°, II°, III°) compared to the control group (C): (**a**) activity of catalase—CAT; (**b**) inhibition of the DPPH radical. The data are presented as means from independent measurements ± standard error. Bars with a different letter indicate significant differences according to HDS Tukey’s test (*p* < 0.05 was accepted as statistically significant). Homogeneous groups are marked with the same letters.

**Figure 2:**
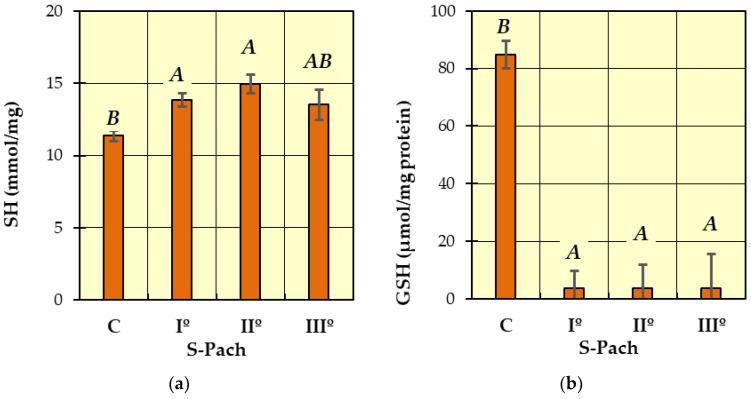
The concentrations of measured antioxidative parameters in the serum of patients with CO poisoning (S-Pach = I°, II°, III°) compared to the control group (C): (**a**) SH; (**b**) GSH. The data are presented as means from independent measurements ± standard error. Bars with a different letter indicate significant differences according to HDS Tukey’s test (*p* < 0.05 was accepted as statistically significant). Homogeneous groups are marked with the same letters.

**Figure 3:**
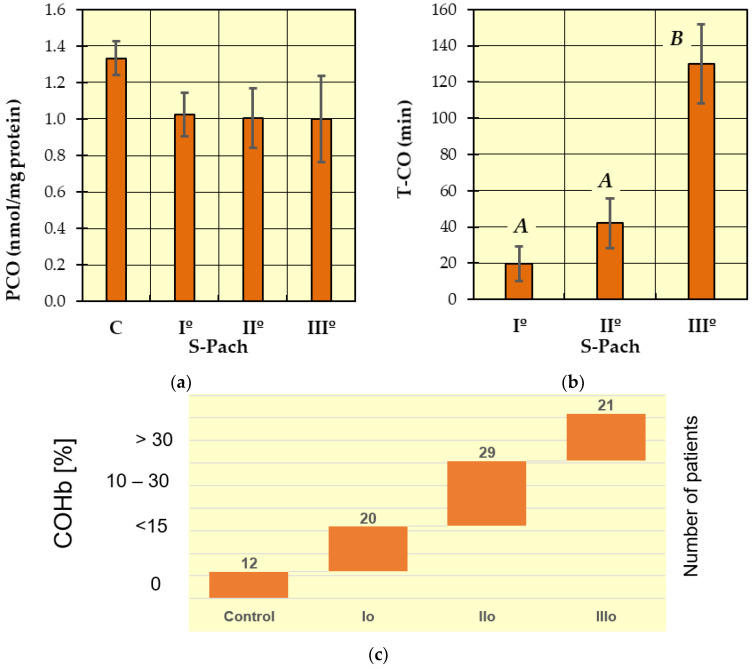
The concentrations of measured antioxidative parameters in the serum of patients with CO poisoning (S-Pach = I°, II°, III°) compared to the control group (C): (**a**) Carbonyl derivates (=CO derivates); (**b**) exposure time of CO. The data are presented as means from independent measurements ± standard error. (**c**) The range of COHb levels for the different Pach scores. Bars with a different letter indicate significant differences according to HDS Tukey’s test (*p* < 0.05 was accepted as statistically significant). Homogeneous groups are marked with the same letters.

**Figure 4:**
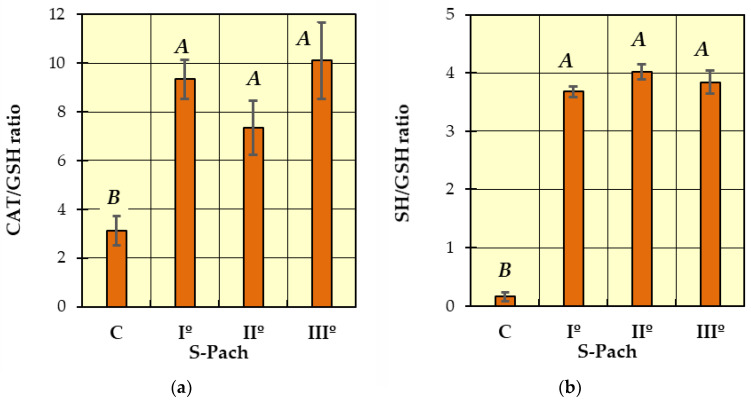
Comparison of patients with CO poisoning (S-Pach = I°, II°, III°) and the control group (C): (**a**) CAT/GSH ratio; (**b**) SH/GSH ratio. The data are presented as means from independent measurements ± standard error. Bars with a different letter indicate significant differences according to HDS Tukey’s test (*p* < 0.05 was accepted as statistically significant). Homogeneous groups are marked with the same letters.

Post hoc Tukey’s tests were performed to identify homogeneous groups for the dependent variables, for which ANOVA allowed rejection of the null hypothesis. In Figure 1, Figure 2, Figure 3 and Figure 4, homogeneous groups are marked with the same letters. According to Tukey’s test, different letters indicate significant differences between the groups (*p* < 0.05).

In Figure 1a (catalase activity—CAT), two homogeneous groups were identified: A (the patients with carbon monoxide poisoning—S-Pach = I°, II°, III°) and B (the patients without carbon monoxide poisoning—control group C). The analysis of catalase activity in the blood serum showed a statistically significant decrease in enzyme activity in all patients with carbon monoxide poisoning (S-Pach = I°, II°, III°) compared to the control group (the patients without carbon monoxide poisoning). The average values of catalase enzyme activity in people with carbon monoxide poisoning were about six times lower compared to the control group.

In Figure 1b (DPPH), two homogeneous groups were identified: A (the patients with carbon monoxide poisoning—S-Pach = I°, II°, III°) and B (S-Pach = C, III°). Compared to the control group, a statistically significant increase in the inhibition of the DPPH radical was observed in the patients with the CO poisoning group (S-Pach = I°, II°). There were no significant differences between the level of DPPH and the severity of the poisoning (S-Pach = I°, II°, III°).

In Figure 2a (SH), two homogeneous groups were identified: A (the patients with carbon monoxide poisoning—S-Pach = I°, II°, III°) and B (S-Pach = C, III°). There were no significant differences between the level of SH and the severity of the poisoning (S-Pach = I°, II°, III°).

In Figure 2b (GSH), two homogeneous groups were identified: A (the patients with carbon monoxide poisoning—S-Pach = I°, II°, III°) and B (the patients without carbon monoxide poisoning—control group C). The level of reduced glutathione was significantly elevated in the control group compared to all patients with carbon monoxide poisoning groups. In all degrees of the Pach scale, the level of reduced glutathione in blood serum was close to zero, while in the control group, it fluctuated between 80 and 90 μmol/mg protein.

The level of carbonyl groups (Figure 3a) was highest in the control group compared to the patients with carbon monoxide poisoning, but the differences are not statistically significant.

In Figure 3b (T-CO), two homogeneous groups were identified: A (S-Pach = I°, II°) and B (S-Pach = I°, II°). Based on data from the literature and clinical experience, we statistically confirmed that the severity of poisoning increased with increasing duration of exposure to carbon monoxide.

In Figure 4a (CAT/GSH ratio) and Figure 4b (SH/GSH ratio), two homogeneous groups were identified: A (the patients with carbon monoxide poisoning—S-Pach = I°, II°, III°) and B (the patients without carbon monoxide poisoning—control group C). Compared to the control group, a statistically significant increase in the CAT/GSH ratio was observed in the patients with carbon monoxide poisoning (Figure 4a). The SH/GSH ratio showed more remarkable differences (Figure 4b)—the values were more than 15 times higher in the patients with CO poisoning compared to the control group. There was no statistically significant difference between the CAT/GSH ratio, SH/GSH ratio and the degree of severity of poisoning patients (S-Pach = I°, II°, III°).

Blood COHb level is well recognized as a biomarker for determining CO poisoning exposure and its effects. Although this is an essential parameter in routine toxicological diagnostics practice, more is needed to provide a complete picture of the biochemical disturbances at the cellular and tissue levels in the case of acute CO poisoning. At this time, it is difficult to explain all clinical problems related to the course of CO poisoning, treatment, and outcome [17,18]. Despite that, the pathophysiology and clinical findings of acute CO poisoning have been extensively reviewed, and many factors contribute to the severity of the poisoning and its outcome. There is still more scientific evidence on the relationship between CO poisoning and parameters related to oxidative stress. CO poisoning is a dynamic pathophysiological process, starting from the hypoxic phase and then reoxygenation with postischemic reperfusion injury. At the tissue level, mitochondrial activity requires oxygen for aerobic ATP synthesis for cellular activity. Aerobic metabolism needs the oxygen cascade, which means the gradient from the atmosphere at sea level (PaO_2_ = 159 mmHg) to the cell cytoplasm (PO_2_ = 4–22 mmHg). Many factors in this cascade affect final mitochondrial PO_2_, including alveolar gas exchange, oxygen transport in the blood, tissue perfusion, tissue type, and local metabolic activity [19]. Under the pathological conditions of CO poisoning, any change in any of the steps in this cascade can result in hypoxia at the mitochondrial level and the formation of oxidative stress.

Many reports have described that when PO_2_ is high in tissues, the generation of ROS, such as hydrogen peroxide and superoxide, increases, causing oxidative stress and free radical damage [19,20,21,22]. It has also been emphasized that ROS cause reperfusion injury during the reoxygenation phase after exposure to CO by decreasing the brain catalase activity that demonstrates hydrogen peroxide production. There was also a decrease in the ratio of reduced/oxidized glutathione, which was quickly corrected by hyperbaric oxygen as opposed to normobaric oxygen, under which it continued to decrease during the first 45 min [23]. Another possible mechanism of ROS generation is the inhibition of xanthine oxidase by allopurinol, which reduces the degree of lipid peroxidation [24]. Most clinical practice guidelines in uncomplicated CO intoxication recommend 100% oxygen therapy until normalization of COHb blood level and 50% oxygen in the next 6 h, and 30% oxygen until 24 h after interruption of CO exposure. Oxygen toxicity is more likely when a non-rebreathing mask (oxygen mask with reservoir) reaches concentrations higher than 60% [19,22,25]. It should be emphasized that in our study, patients received 100% oxygen therapy for an average of 30 to 60 min at the time of blood sampling. Such iatrogenic hyperoxia induces the formation of free radicals, which could be additive to the free radicals generated in CO poisoning. There is a lack of data on the direct or indirect impact of a very high concentration of free oxygen on antioxidative blood parameters. Many complex antioxidant mechanisms offer protection against peroxidation, involving many molecular pathways and molecules. Antioxidant molecules are not independent, and variable biochemical integration can be observed between different antioxidants. In our study, to evaluate oxidative stress and antioxidative defense (scavenger) systems, we used in vivo analyses that included the detection of blood biomarkers such as free radicals (DPPH), sulfhydryl (thiol) and carbonyl groups as a marker of protein damage, non-enzymatic antioxidants (reduced glutathione), and enzymatic antioxidants (catalase). Considering the limitations concerning the sensitivity, specificity, and timing of ROS and antioxidative biomarker analysis, we propose two main theses for our study.

(1) In the case of acute CO poisoning, ROS production with free radicals, increased consumption and/or reduced stores of antioxidants, and/or decreased activity of antioxidants [14,26,27,28,29].
(2) During intense treatment of CO poisoning with oxygen therapy at very high concentrations of free oxygen, it is suspected that the antioxidant systems are eventually overwhelmed, and the rate of cell damage exceeds the capacity of the systems that prevent or repair it.

The results showed that the antioxidant parameters in CO-poisoned patients changed. Two primary changes were observed: a reduction in CAT activity and a decreased level of reduced GSH. These parameters are more closely related to changes in cellular antioxidant status. SOD eliminates superoxide radicals (O, O_2_●) by converting them into oxygen and hydrogen peroxide (H_2_O_2_). CAT and glutathione peroxidase (GPx) are direct antioxidant enzymes that decompose hydrogen peroxide into oxygen and water [30,31]. The pro-oxidant activity of H_2_O_2_ is due to its reduction by one electron in hydroxyl radical (OH). The formation and reactions of (OH) are associated with chemical, non-enzymatic reactions, which are beyond any cellular control or antioxidant defense mechanism. These processes are known as Haber-Weiss and Fenton reactions [32].

CAT decomposes hydrogen peroxide at high rates but with low affinity. It is most useful when H_2_O_2_ production or accumulation peaks are presented. The lack of catalase (acatalasemia) increases oxidative stress and induces human pathologies [20,33]. According to the Pach scale, the mean reduction in CAT observed in CO-poisoned patients during the first hour was six times lower than in the controls and was not correlated with the severity of CO poisoning. There are few data on the influence of CO and oxygen toxicity on the activity of antioxidant enzymes. For this reason, comparing our results with those of the other authors’ reports is a challenge. CAT has been suggested to play its role when the glutathione peroxidase (Gpx) pathway reaches saturation with the substrate (H_2_O_2_). Gpx slowly decomposes H_2_O_2_ but with higher affinity, so it is most beneficial to decompose the small amounts of hydrogen peroxide produced under physiological conditions inside cells. Gpx needs a cofactor like reduced GSH or another sulfhydryl(thiols) to function. In the present study, we did not measure the activity of Gpx, but we observed a decrease in the level of reduced glutathione (GSH), which Gpx probably used to decompose H_2_O_2_. The increased peaks of H_2_O_2_ with Gpx saturation and CAT saturation could explain the low CAT activity observed in our study. Glutathione also acts as a cofactor for glutathione S-transferases (GSTs), enzymes of phase II metabolism. GSTs conjugate glutathione with other electrophilic compounds and lead to the depletion of reduced glutathione (GSH) [34]. The increased ratios of CAT/GSH and SH/GSH obtained in our study confirm the lack of reduced GSH in the CO-poisoned group. Both calculated ratios significantly differentiate CO-poisoned patients from the control group but did not show statistical significance in determining the stage of CO poisoning.

Another statistically significant change was observed due to the sulfhydryl (thiol) groups (–SH), an increased level in all CO-poisoned patients, irrespective of the severity of the poisoning. In fact, in the case of oxidative stress, the oxidation of cellular and extracellular proteins sulfhydryl (thiol) groups occurs directly due to ROS. Oxidation of the sulfhydryl (thiol) group alters the tertiary structure of many structural and functional proteins, causing their inactivation and aggregation. It may also lead to impairment of calcium homeostasis with calcium accumulation in the cytosol and increased activation of calcium-dependent proteases and phospholipases [24]. A high SH/GSH ratio may also indicate a significant rise in the sulfhydryl (thiol) group. Many studies show that the assessment of the content of sulfhydryl (thiol) groups serves as a better indicator of oxidative stress than measuring the total oxidative state [35,36,37]. On the other hand, some data show that acute oxygen toxicity during CO treatment is mainly due to the oxidation and polymerization of the -SH groups of enzymes that lead to their inactivation.

No statistically significant changes were observed in the carbonyl groups, reflecting protein oxidations. The difference was insignificant due to the relatively small number of patients with CO poisoning. Including more significant CO-poisoned patients in the study would increase the statistical significance of this parameter.

Next, increased (at statistical significance) inhibition of the DPPH radical was observed in the CO-poisoned group. According to the Pach scale, this increase in DPPH inhibition was independent of the severity of CO poisoning. This observation is difficult to interpret because DPPH (1,1-diphenyl-2-picryl hydrazyl), a free radical, can react with antioxidant and oxidative molecules. According to the Pach scale, a positive correlation was also found between the time of exposure and the severity of CO poisoning; despite significant variability, the trend remains clear.

In carbon monoxide patients, the lungs, hearts, and brain are often affected and cause coma and death in severe poisoning, resulting in immediate and delayed neuronal damage in some brain regions that cannot be easily explained by tissue poisoning. One possibility is that cell injuries during and after CO poisoning are related to brain reactions to oxygen species. Exposure to CO can cause a variety of perivascular processes, including oxidative stress that activates *N*-methyl-D-aspartate (NMDA) and nitrate oxide synthase (nNOS) in neurons. These events are essential for the progression of CO-mediated neuropathology. It follows from the experimental data that the poisoned patients had a significant increase in level due to thiol groups and the 2,2-diphenyl-1-picryl-hydrazyl radical, with no significant differences according to the severity of poisoning. The catalase-to-glutathione and thiol-to-glutathione ratios showed the most important differences between the poisoned patients and the control group, with a significant increase in the poisoned group. The ratios did not differentiate the severity of the poisoning. The carbonyl level was highest in the control group compared to the poisoned group but was not statistically significant. Our critical assessment is that it is challenging to use oxidative-stress-related parameters to evaluate the severity of CO poisoning, the outcome and treatment options. This is the first critical observation compared to the widespread belief in the literature that such relationships occur. However, careful statistical analysis revealed that the problem is more complex, and the discussion cannot be led without additional research. Thus, it should be noted that further studies are required, focusing on molecular mechanisms.

## 3. Materials and Methods

### 3.1. Materials

**Figure 5:**
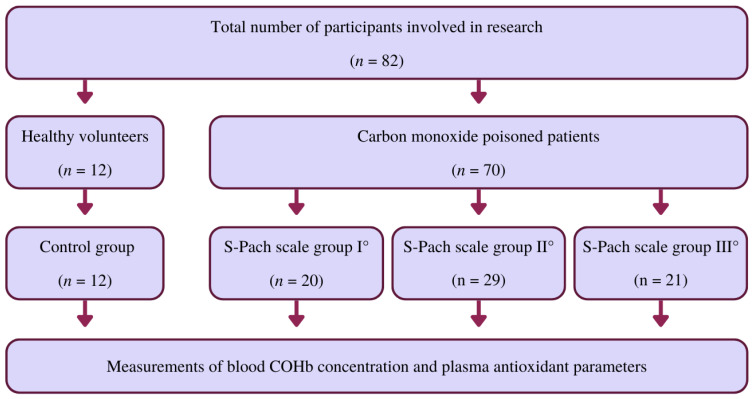
Flow chart showing the division of research participants into the control group and also the groups of CO-poisoned patients according to the S-Pach scale.

Our studies involved patients (*n* = 82, age range: 23–43 years, 51.2% females, 48.8% males) admitted to the Clinical Department of Toxicology of Jagiellonian University Medical College (Kraków, Poland) during 2015–2020. For the control group, we chose 12 healthy volunteers (31–63 years old). The control group comprised six women (mean age = 46 years) and six men (mean age = 47 years). The control group consisted only of healthy volunteers who were non-smokers with no digestive problems (e.g., malnutrition, obesity). The poisoned patients involved (*n* = 70) were divided into three groups according to the S-Pach scale: I°, II°, III°. The study group consisted of patients with confirmed CO poisoning who were included in our study without any exclusions, especially without other exposures to other xenobiotics. In both groups of patients, the antioxidant parameters in the plasma were measured. The characteristics of the research participants, taking into account their age, sex, and the group to which they were assigned, are presented in Supplementary Materials (Table S1). Poisoning was diagnosed based on medical history, clinical symptoms, and COHb blood level. Blood samples to determine COHb were taken immediately after admission to the emergency department, and COHb analysis was performed using point-of-care (POCT) and blood gas analyzers. The Department of Toxicology is located in the main city center of Krakow. According to emergency medicine principles, an ambulance should reach the accident site (a patient with CO poisoning) in 7 min. Within 15 min, it should contact the nearest emergency department. Thus, our patients were admitted to the emergency department up to 30 min after the termination of CO exposure. Patients received oxygen during transport to the hospital. Whole blood was collected in anticoagulant-treated tubes (EDTA treated). To obtain plasma, cells were removed by centrifugation in a refrigerated centrifuge for 10 min at 1500× *g*. Platelets were then depleted from the plasma sample by centrifugation for 15 min at 2000× *g*. The plasma was then immediately transferred to a clean polypropylene tube after centrifugation. During handling, the plasma samples were kept at −2 to 8 °C. Acute CO poisoning was diagnosed based on medical history, particularly the source and time of exposure to CO, clinical symptoms, and toxicological laboratory parameters. Exposure time to CO was calculated based on data obtained during the medical history of patients and family members. In addition, the information contained in the rescue operation report prepared by paramedics was also based on information. The Pach scale (see Table 1) was used to assess the severity of the poisoning. The flow chart shows the research participants’ division into the control group and the CO poisoning patients’ groups according to the S-Pach scale (Figure 5).

### 3.2. Measurement of Oxidative-Stress-Related Parameters

#### 3.2.1. Measurement of Catalase (CAT)

Catalase (CAT; EC 1.11.1.6) activity in plasma samples was measured following the kinetic method described by Aebi [38]. Changes in absorbance from the beginning to the end of the reaction were measured at a wavelength of 240 nm. The enzymatic activity was calculated as U/mL of plasma. In this case, one unit of CAT activity was defined as the enzyme that decomposes 1 mol of H_2_O_2_ per minute. Catalase activity was expressed in U/mg protein.

#### 3.2.2. Measurement of 2,2-Diphenyl-1-picryl-hydrazyl (DPPH)

Plasma antioxidant properties were measured using the DPPH test based on the method reported by Molyneux and modified by Annegowda [39,40]. In general, DPPH, in its stable radical form, absorbs at 517 nm, but its absorption decreases upon reduction by an antioxidant in a sample. A 0.6 mM solution of 2,2-diphenyl-1-picryl-hydrazyl (DPPH) was prepared in methanol. All plasma samples were deproteinated with 10% TCA (trichloroacetic acid) and centrifuged (Micro 200 R) at 4 °C and 2500× *g*. The percentage of DPPH in the supernatant obtained was measured at 30 min. All samples were measured in triplicate. The absorption of the control sample (a DPPH methanol solution) was measured at the beginning and end of the analysis. The percentage of inhibition of DPPH was calculated using the following formula. DPPH % = [(ADPPH − A30 min)/ADPPH] × 100, where A30 min is the mean absorbance of the sample, and ADPPH is the absorbance of the control sample.

#### 3.2.3. Sulfhydryl Groups (-SH)

The total sulfhydryl content was determined using Ellman’s method with some modifications [41]. The absorbance was measured at 412 nm, and the SH content was calculated using a molar extinction coefficient of 13,600 M^−1^ cm^−1^. The -SH group content was expressed in mmol/mg protein.

#### 3.2.4. Measurement of Reduced Glutathione (GSH)

Reduced glutathione levels were determined in blood plasma after deproteination with trichloroacetic acid (TCA). The free -SH groups were determined using the Ellman method in the supernatant obtained, and the GSH content was expressed in the GSH µmol/mg protein [41].

#### 3.2.5. Measurement of the Carbonyl Group (=CO Derivates)

The protein carbonyl group contents were measured using the method described by Levine et al. [42]. The absorbance was measured at 370 nm. The carbonyl group content (=CO derivates) was expressed in nmol/mg protein.

### 3.3. Statistical Analysis

The antioxidative parameters of serum (catalase—CAT, DPPH, total sulfhydryl contents—SH, reduced glutathione levels—GSH, protein carbonyl groups—=CO derivates) were evaluated for 82 patients divided into four groups: C control group, Pach scale I°, Pach scale II°, and Pach scale III°. Using Dixon calculations, the outliers’ results were removed (a total of eight measurements) [43]. The statistical hypothesis that the impact of carbon monoxide poisoning severity on antioxidative parameters is significant was verified using an analysis of variance (ANOVA) [44,45,46,47,48,49,50,51]. Preliminary analysis (two-factor ANOVA) showed no statistically significant differences in dependent variables between men and women. For this reason, one-way ANOVA was performed to verify the null hypothesis. ANOVA was conducted for each of the following dependent variables: CAT, DPPH, SH, GSH, =CO derivates, quotient CAT and GSH (CAT/GSH), quotient SH and GSH (SH/GSH), and time of exposure to carbon monoxide (T-CO). The Pach scale (S-Pach) was the intra-group factor. The S-Pach was analyzed on four levels: control—C (the patients without carbon monoxide poisoning), I°, II°, and III° (the patients with carbon monoxide poisoning). If the null hypothesis is rejected based on the ANOVA results (no significant differences between the groups), post hoc tests (multiple comparison tests) must be performed. To determine homogeneous groups, the honest significant difference (HSD) of Tukey’s test was used [44,45,51]. Data were analyzed using the STATISTICA v.14.0 software (Dell Inc. (Tulsa, OK, USA, 2016)), Dell Statistica (data analysis software system), version 13 (software.dell.com). Values of *p* < 0.05 were considered statistically significant.

## 4. Conclusions

In conclusion, the results emphasized the need to observe the redox status of CO-poisoned patients in the acute phase of intoxication and after the discontinuation of therapy. Differences in antioxidant status are difficult to explain due to the diversity of patients in the study group. Therefore, after critical evaluation, it can be concluded that it is challenging to use oxidative-stress-related parameters to evaluate the severity of CO poisoning, outcome, and treatment options. Compared to the literature [28], a correlation is observed only with a few parameters. To our knowledge, this is the first critical observation to date compared to the widespread belief in the literature that such relationships occur. Many variables affect the course and severity of CO poisoning, such as exposure time, age, comorbidity, personal sensitivity, COHb level, and oxygen therapy. More detailed observations on antioxidant status could only be collected in experiments conducted on animal models under more strictly controlled conditions. Recognition of these relationships allows for a proper approach to CO poisoning with oxygen therapy and antioxidant use. However, careful statistical analyses reveal that the problem is more complex and that discussions cannot be conducted without additional research. Therefore, further research is necessary, focusing on the molecular mechanism.

## Figures and Tables

**Table 1:** Pach scale—table for calculation of CO poisoning severity in Poland.

Points	0	1	2	3
Age (years)	<29	30–39	40–49	>50
CO exposition time (min)	<30	31–60	61–120	>120
Neurological injury degree	I° - No consciousness disturbances and other neurological changes in physical examination.	II° - Consciousness disturbances (somnolence and agitation); - Hyperreflexia; - Positive Babinski reflex; - Tonic–clonic convulsions; - Increased muscular tone.	III° - Unconsciousness.	IV° - Loss of consciousness; - Hyperreflexia; - Positive Babinski reflex; - Tonic–clonic convulsions; - Decreased muscle tone; - Bradyreflexia.
COHb serum level (%)	0	<15	15–30	>30
Lactate serum level (mmol/L)	1.0–1.78	1.8–3.6	3.7–5.4	>5.5
The severity of CO poisoning: I° light 1–4 points, II° medium 5–8 points, III° sever > 9 points				

**Table 2:** One-way ANOVA results for catalase (CAT), DPPH, sulfhydryl (SH), reduced glutathione (GSH), protein carbonyl group (=CO derivates), quotient CAT and GSH (CAT/GSH), quotient SH and GSH (SH/GSH), and time of exposure to carbon monoxide (T-CO).

	Source of Variation	SS ^1^	df ^1^	MS ^1^	F ^1^	*p* ^1^	Significant
CAT	S-Pach ^2^	530530	3	176843	50.194	<0.001	Yes
DPPH	S-Pach	8352	3	2784	15.597	<0.001	Yes
SH	S-Pach	169.1	3	56.4	10.338	<0.001	Yes
GSH	S-Pach	132439	3	44146	52.231	<0.001	Yes
=CO derivates	S-Pach	2.051	3	0.684	2.030	0.117	No
CAT/GSH	S-Pach	701.6	3	233.9	15.929	<0.001	Yes
SH/GSH	S-Pach	269.3	3	89.8	404.347	<0.001	Yes
T-CO	S-Pach	40945	2	20473	10.794	<0.001	Yes

^1^ SS—the sum of squares between groups, df—the number of degrees of freedom, MS—the mean sum of squares between groups, F—the test statistic value, *p*—probability. ^2^ S-Pach—Pach scale.

**Table 3:** Results of antioxidant parameters for different groups (value of S-Pach): catalase (CAT), DPPH, sulfhydryl (SH), reduced glutathione (GSH), protein carbonyl group (=CO derivates), quotient CAT and GSH (CAT/GSH), quotient SH and GSH (SH/GSH), and time of exposure to carbon monoxide (T-CO). Data are presented as means from independent measurements ± standard deviation (SD).

	Control	I°—Pach	II°—Pach	III°—Pach
CAT (U/mg protein)	196 ± 82.2	35.0 ± 18.3	27.3 ± 9.6	37.8 ± 22.4
DPPH (%)	5.9 ± 6.5	23.9 ± 16.6	30.1 ± 20.5	25.6 ± 11.4
SH (mmol/mg protein)	11.4 ± 2.4	13.8 ± 2.6	14.9 ± 1.6	13.5 ± 1.1
GSH (μmol/mg protein)	84.8 ± 41.9	3.77 ± 0.2	3.73 ± 0.16	3.75 ± 0.14
=CO derivates (nmol/mg protein)	1.333 ± 0.57	1.025 ± 0.58	1.005 ± 0.58	1.001 ± 0.66
CAT/GSH ratio	3.12 ± 2.96	9.34 ± 4.88	7.35 ± 2.62	10.1 ± 6.08
SH/GSH ratio	0.158 ± 0.086	3.678 ± 0.692	4.022 ± 0.523	3.842 ± 0.711
T-CO (min)	-	19.8 ± 7.0	42 ± 37	130 ± 125.7

## Data Availability

The datasets generated during and/or analyzed during the current study are available from Kamil Jurowski (toksykologia@ur.edu.pl) upon reasonable request.

## Supplementary Materials

The supporting information can be downloaded at: https://www.mdpi.com/article/10.3390/ijms241310784/s1.
