# Supplementary material for: The Critical Assessment of Oxidative Stress Parameters as Potential Biomarkers of Carbon Monoxide Poisoning
Source: Int J Mol Sci. 2023 Jun 28;24(13):10784. doi: 10.3390/ijms241310784 (PMC10341785; doi:10.3390/ijms241310784)
Supplement: Supplementary file 1 [file ijms-24-10784-s001.zip › ijms-2323583-supplementary.pdf]

Table S1. Characteristics of research participants.

Patients number	Age	Gender (F/M)	Pach scale	Patients number	Age	Gender (F/M)	Pach scale	Patients number	Age	Gender (F/M)	Pach scale
**1**	56	F	Control	**29**	47	M	II^0	**57**	37	F	Control
**2**	32	M	I^0	**30**	68	M	III^0	**58**	62	F	III^0
**3**	55	F	III^0	**31**	51	M	II^0	**59**	23	F	II^0
**4**	65	F	III^0	**32**	23	M	I^0	**60**	41	M	II^0
**5**	24	F	I^0	**33**	55	F	III^0	**61**	52	M	III^0
**6**	38	F	Control	**34**	26	M	I^0	**62**	23	M	I^0
**7**	19	F	II^0	**35**	29	F	I^0	**63**	63	M	Control
**8**	29	M	II^0	**36**	47	F	II^0	**64**	30	M	II^0
**9**	31	M	II^0	**37**	40	M	II^0	**65**	28	F	I^0
**10**	28	F	I^0	**38**	68	M	III^0	**66**	24	F	I^0
**11**	69	F	III^0	**39**	54	F	Control	**67**	41	F	II^0
**12**	56	M	Control	**40**	36	F	I^0	**68**	28	M	I^0
**13**	67	F	III^0	**41**	53	M	III^0	**69**	68	F	III^0
**14**	42	M	Control	**42**	34	F	II^0	**70**	31	M	Control
**15**	61	F	III^0	**43**	50	M	III^0	**71**	47	F	II^0
**16**	44	M	II^0	**44**	41	M	II^0	**72**	52	F	III^0
**17**	29	M	I^0	**45**	50	F	II^0	**73**	24	F	I^0
**18**	58	M	III^0	**46**	56	F	III^0	**74**	69	M	III^0
**19**	61	F	III^0	**47**	19	M	I^0	**75**	46	M	II^0
**20**	31	M	I^0	**48**	41	F	Control	**76**	25	F	I^0
**21**	55	F	II^0	**49**	39	M	II^0	**77**	58	M	Control
**22**	45	F	II^0	**50**	55	M	III^0	**78**	58	F	III^0
**23**	40	M	II^0	**51**	32	M	Control	**79**	46	F	II^0
**24**	36	F	I^0	**52**	61	F	III^0	**80**	24	F	I^0
**25**	45	F	II^0	**53**	25	F	I^0	**81**	27	M	I^0
**26**	43	M	II^0	**54**	47	M	II^0	**82**	33	M	II^0
**27**	47	F	Control	**55**	29	F	II^0				
**28**	59	M	II^0	**56**	46	M	II^0				
